# On using centrality to understand importance of entities in the Panama Papers

**DOI:** 10.1371/journal.pone.0248573

**Published:** 2021-03-25

**Authors:** Mayank Kejriwal

**Affiliations:** Information Sciences Institute, University of Southern California, Marina del Rey, CA, United States of America; Unviersity of Burgundy, FRANCE

## Abstract

The Panama Papers comprise one of the most recent influential leaks containing detailed information on intermediary companies (such as law firms), offshore entities and company officers, and serve as a valuable source of insight into the operations of (approximately) 214,000 shell companies incorporated in tax havens around the globe over the past half century. Entities and relations in the papers can be used to construct a network that permits, in principle, a systematic and scientific study at scale using techniques developed in the computational social science and network science communities. In this paper, we propose such a study by attempting to quantify and profile the importance of entities. In particular, our research explores whether intermediaries are significantly more influential than offshore entities, and whether different centrality measures lead to varying, or even incompatible, conclusions. Some findings yield conclusions that resemble Simpson’s paradox. We also explore the role that jurisdictions play in determining entity importance.

## Introduction

Since being leaked in 2015, the so-called Panama Papers (an 11.5 million document trove detailing information on roughly 214,000 offshore entities, intermediaries and officers) exposed corruption, money laundering and tax evasion at an unprecedented global scale. An important economic consequence of this leak, according to a recent report, has been the collection of more than 1.2 billion USD in back taxes and penalties by governments around the world [1].

Because the condensed, publicly available version of the data can be expressed as a graph, structural properties of the entities can be quantified using network science. Particularly interesting is the question of which entities are *influential* in such a network, and to what extent the importance is determined by factors such as the *class* of the entity (e.g., whether the entity is an intermediary, an offshore organization or an officer of the organization or intermediary), the *computational measure* employed for quantifying the importance (e.g., the betweenness centrality [2]) and the *national jurisdiction* affiliated with the entity (e.g., Hong Kong).

In this paper, we use the data made publicly available by the International Consortium of Investigative Journalists (ICIJ) to selectively construct networks and study importance of entity classes in the Panama Papers by modeling entities as vertices. An established way to understand which vertices are focal or important is by computing their *centrality*. Since being published more than a half-century ago, centrality metrics like betweenness and information centralities are well-studied and established in network science [2, 3].

However, it has also been understood as early as the 1970s [4] that different centrality measures seem to underlie different real-world social phenomena. In the context of the Panama Papers then, several important questions arise. For instance, given a centrality metric, which class of entities is the most ‘influential’ on average? Are there strong, positive correlations when either the class or the centrality measure is varied? How important is the role of an entity’s jurisdiction in determining whether it is focal? Thus far, these questions have not been answered using a well-defined quantitative methodology for the entities in the Panama Papers.

We design and conduct a rigorous series of experiments to answer these questions, while also illuminating interesting aspects of different centrality measures such as betweenness and current flow. For instance, our experiments show that, while some findings are consistent across almost all centrality measures (e.g., high scores are typically assigned to intermediaries by almost all centrality measures), there are significant distributional and statistical disparities between centralities (and in particular, the information centrality), especially when conditioned on an entity class. Some findings are also found to lead to results that resemble *Simpson’s paradox* [5], especially when comparing the findings on a particular entity class to the overall network.

We also qualify some of our findings while controlling for *national jurisdiction*, and find intriguing relationships between the different centrality measures and entity classes even at the aggregate level of jurisdictions. Our full set of results provides detailed insights on the distribution of centrality in an interconnected system of entities that, despite having attracted significant qualitative scrutiny from legal scholars and sociologists [6, 7], has received little attention (especially at scale) from the computational social sciences.

## Background and related work

Since the release of the Panama Papers by ICIJ, multiple analyses have been presented, including a bestselling book [1]. Much of this analysis has been sociological or legal in nature. For example, [8] discuss how firms use secret offshore vehicles to ‘finance corruption, avoid taxes and expropriate shareholders’. In a law review, [6] study the disclosures surrounding the leaked documents and provide a discussion on the impact of bribery on the global community, as well as tax evasion. Numerous other references cover similar issues, often spanning disciplines: a selected few include [7, 9–11]. Computational studies of any kind have not been common; [12] is one rare example of a work that uses the network to study the financial networks of the Middle East, but the scope and analysis is both geopolitically and structurally limited. Another work, which involves information extraction but not network science, is the multilingual system proposed in [13]. In our own recent work [14], we did structural studies on selectively constructed Panama Papers networks by using network science, and found that the networks tend to follow a power-law degree distribution, but are extremely fragmented. However, the importance of entities, or dependence of any such importance on the entities’ jurisdictions, were not studied in that work.

Another relevant paper, very recently published [15], proposed an algorithm to find ‘suspicious’ entities in databases such as this one, where suspicious entities were defined as entities that were likely to engage in illegal acts. The authors of that work used external databases and known lists of suspicious entities to verify their ground truth. In contrast, this work makes no claim of the legality of actions, but is attempting an aggregate study of entity importance, after adopting appropriate controls, using structural properties of the constructed networks.

Centrality is an extremely well-studied area in network science, and the first centrality metrics were published more than fifty years ago [2]. Analysis and quantification of vertex (and in some cases, link) importance using centrality is standard in computational network science [3], but many questions remain (regarding both practice and theory), especially involving networks that are not as ubiquitous or well-studied as social networks [3]. Furthermore, there is no one ‘good’ centrality measure; several decades earlier, Freeman did detailed studies on centrality and suggested that different centrality measures correspond to different social occurrences [4]. The work by Freeman is particularly relevant for this paper since we also provide reasonably strong evidence that different centrality measures seem to correspond to different phenomena in the network underlying the Panama Papers, and in some cases, exhibit interesting aspects such as Simpson’s paradox (a phenomenon which Freeman did not explore in his own studies) [5]. More recently, Amrit and Maat [16] study information centrality (one of the centralities we also employ in this work as a measure of importance) in a simulated setting and show that, contrary to previous work that postulated that it was more correlated with closeness centrality, it is more similar to degree and eigenvector centrality. Our data in the real-world Panama Papers setting partially support this conclusion, as we discuss subsequently, though we find that the conclusion can change depending on the class of entity being studied.

In other work, Abbasi et al. [17] use a similar kind of argument to hypothesize that the degree centrality of a researcher’s collaboration network positively correlates to performance. Other papers have used other centrality measures to study diverse phenomena; for a detailed (and relatively recent) survey on centrality and its history, as well as applications, we recommend the work by Das et al. [3]. Similarly, Lu et al provide a review on vital nodes identification in complex networks, including an introductory treatment of the various centralities used in this article. [18]. Ghalmane et al. [19] extended the standard centrality measures, including betweenness and closeness, that were originally defined for networks with no community structure to modular networks. Their proposed “modular centrality” is a two-dimensional vector. Later, the modular centrality was extended to networks with overlapping communities [20]. Sciarra et al. propose multi-component centrality metrics as a natural extension of standard centrality metrics by using tests on a variety of networks to show that standard metrics can perform less than satisfactorily [21]. Rajeh et al. [22] study the interplay between hierarchy and centrality in complex networks; in particular, their results show that network density and transitivity can play an important role in determining the *redundancy* between centrality and hierarchy measures. Many of the centrality measures studied in that article are also used herein for experiments, including the current-flow closeness centrality (which has been shown to be equivalent to information centrality), betweenness and degree centrality. While we do not study hierarchy directly in this work, it remains an interesting area of future research in the context of studying the Panama Papers.

## Research questions

We briefly state the research questions under consideration in this paper below. While the first question studies importance of individual entities in the full (i.e. global) Panama network, the construction of which is technically described in the next section, the second question attempts to understand differences at the level of national jurisdictions.

1. **Class-specific centrality distributions**: Under the assumption that established centrality measures (such as degree and betweenness centrality) can be used to measure node importance, which of the three *entity classes* (intermediaries, offshore entities and officers) have relatively high centrality values? Furthermore, are their centrality distributions consistent and similar across different centrality measures?
2. **Jurisdictional dependencies**: Given that we know the jurisdictions of many of the entities in the network, can we quantify the effect of jurisdictions on the different classes of entities? Do some jurisdictions have higher probability of containing more important entities for a given class (e.g., intermediaries) than others? When using aggregated measures of entity importance as a variable, how strong are the associations between jurisdictions?

## Materials and methods

**Fig 1:**
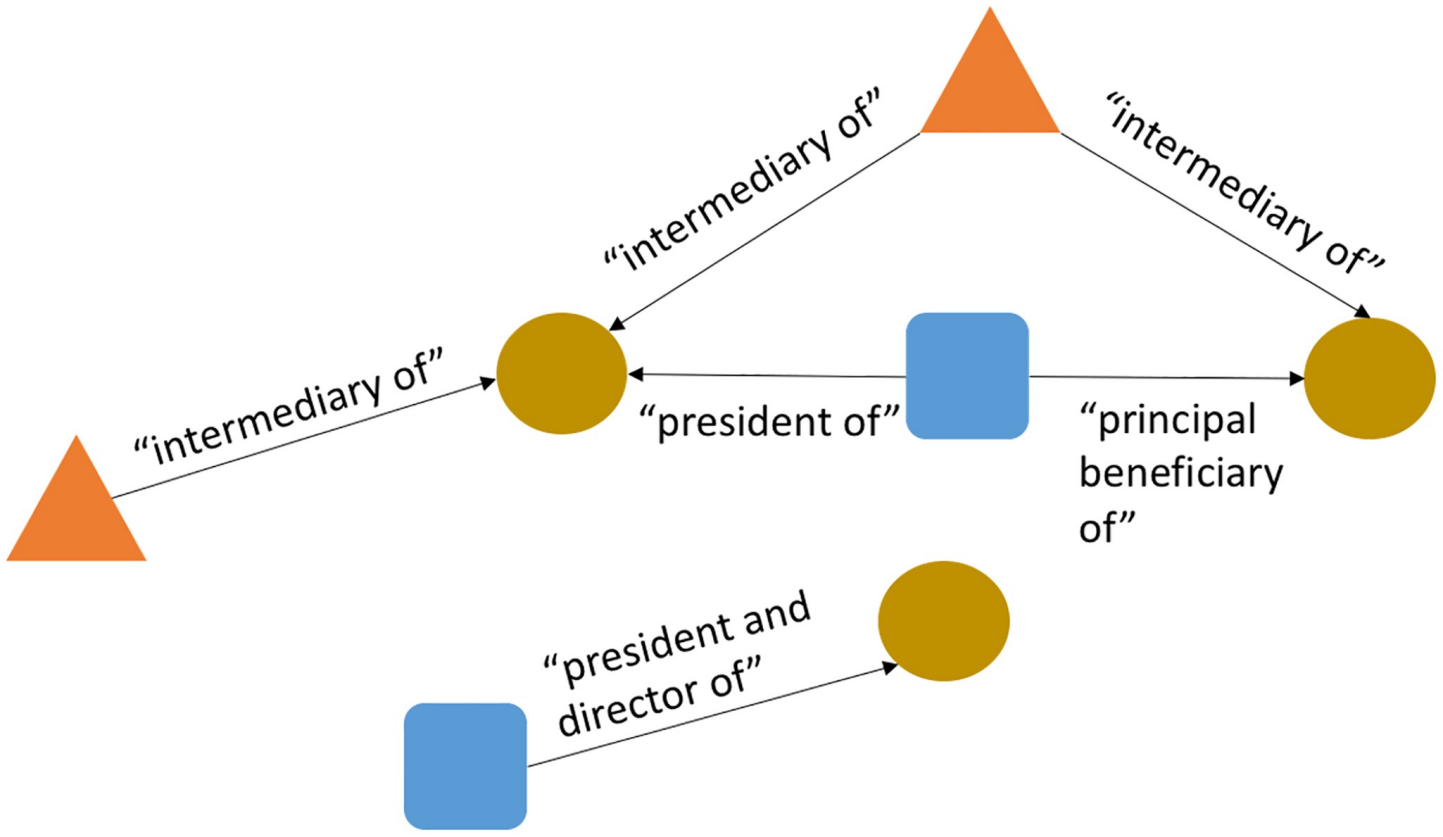
An illustration showing one possible interlinked arrangement of intermediaries (triangles), officers (squares) and offshore entities (circles). We use the actual relations (edge labels) in the dataset to show the manner in which these entities can be linked.

We use the latest version of the Panama Papers dataset available on the ICIJ page [23]. Many other details, including ethical statements on using the data for research as well as definitions on some of the key terms, can also be found on the project page. There are three main *classes* of entities, namely *offshore entities*, *intermediaries*, and *officers* that are of interest to us in this paper and are modeled as vertices in the network on which we conduct centrality studies. A fourth class of ‘entity’ (the string representation of an entity’s *address*) is also present but does not have outgoing edges, and serves no purpose for these studies; hence, we only consider the three entity classes noted above. An offshore entity is defined by the ICIJ as “a company, trust or fund created by an agent in a low-tax jurisdiction that often attracts non-resident clients through preferential tax treatment.” An intermediary is defined as (usually) a “a law-firm or a middleman that asks an offshore service provider to create an offshore firm for a client.” An officer could belong to a “broad class of individuals, including beneficiaries and nominees, who are in a position of significant influence in the associated organization, which is an offshore-entity or an intermediary.” We provide an illustrative visualization in Fig 1. An important point to note is that, despite what the word implies, the term “officer” can legally be used to refer to a corporate entity rather than a human being. Furthermore, as shown in the figure, an intermediary can serve more than one offshore entity.

While the original network has both directionality, and labels on edges, we consider the simple, undirected equivalent of this network. There are two reasons for this decision. First, edge directions are arbitrary in the network and are based on the edge label (e.g., if we changed a relation from ‘president of’ to ‘has president’, the directionality of all edges with this relational label would reverse). As such, directions do not represent a meaningful real-world quantity like information flow or follower/followee semantics, as in other social, supply-chain or organizational networks. Second, not all centrality metrics are defined for directed networks, and in the general case, the centrality metrics have been best studied for simple, unlabeled networks. To obtain a simple, undirected network from the raw data, we ignore the edge labels, remove directions and collapse multiple edges between two vertices into one canonical edge. This resulting network has 657,489 edges and 559,433 non-singleton nodes (a *singleton* node being defined as one that has no edges incident upon it). In studying the degree and connected component (CC) distributions of this network, previous work has found that this network is disconnected, and that the degree and CC size distributions obey the power law. The network also has very low density (≤ 10^−5^) and transitivity (≤ 10^−7^) [14]. Similar findings hold even when the network is constructed in slightly different ways. For example, when only retaining nodes that are incident to an “officer_of” relation (which yields a sub-graph that eliminates the intermediaries in the network), we find that the resulting graph still exhibits low density and transitivity. Analogous findings are observed, when we retain nodes connected through an “intermediary_of” relation instead (which eliminates officers). [14]. In this paper, both research questions are investigated using the single network described above (which contains all three entity types, namely officers, offshore entities and intermediaries), although in researching the second research question, aggregations are conducted at the level of jurisdictional dependencies for a given entity type. For example, when studying intermediaries in Germany, aggregations would be conducted for the measure under study (which is typically a centrality measure, as subsequently discussed) only for intermediaries in Germany. However, this does not involve construction of a new network (involving only intermediaries in Germany, for example). In fact, such an exercise would be counter-productive to the scientific aims of this paper, since the Panama Papers has complicated international linkages that strongly affect the centrality values. It is essential to study the properties (e.g., individual node centralities) of the network in its global context, as we do in this paper. Once computed, the individual metrics can be grouped and aggregated in a variety of ways, depending on the research question being studied.

As first described in the introduction, our goal in this work is to study the importance of various classes of nodes in the Panama Papers. In keeping with prior studies, we also proposed using centrality measures to measure such importance. Below, we enumerate the specific centrality measures used in this paper. All experiments in this paper were conducted using the NetworkX package [24]. Note that both the research questions mentioned earlier rely methodologically on these centrality measures.

1. **Degree centrality**: A conceptually simple measure of centrality, the degree centrality of a node *v* is a function of the node’s degree *deg*(*v*). We obtain a normalized value by dividing each node’s degree *deg*(*v*) by |*V*| − 1 (the maximum theoretical degree).
2. **Information centrality**: Information centrality, originally proposed in 1989 [25], is based on the ‘information’ contained in all possible paths between pairs of points. It was motivated by general ideas of statistical estimation, and departed from many of the traditional centralities (such as betweenness) in considering *all* paths between points rather than just the ‘geodesic’ (or shortest) paths. It was shown to be equivalent to current-flow closeness centrality [26]. For complete details, including the definition of ‘information’ used by the proposing authors, we refer the reader to the original paper by [25].
3. **Closeness centrality**: In a connected graph, the normalized closeness centrality (or closeness) of a node is the average length of the *shortest path* between the node and all other nodes in the graph. Since closeness centrality is only well-defined if the graph is connected, we independently compute it for nodes in each of the individual connected components in *G*. In future studies, one could also consider a variant of closeness centrality where an adjustment, suggested by Wasserman and Faust [27], could be used to account for the imbalanced size of components.
4. **Betweenness centrality**: Betweenness centrality, one of the most classic measures of centrality that was first proposed in 1948 [2], is a measure of the importance of a node over the information flow of information between every node pair assuming that the information primarily flows over the *shortest paths* between the pair. Specifically, betweenness centrality of a node *v* is the sum of the fraction of all-pairs shortest paths that pass through *v*. For a graph with hundreds of thousands of nodes and edges, computing the exact betweenness centrality is not feasible in reasonable time. Hence, we used a well-known approximation method [28], whereby we randomly choose *k* ‘pivot’ nodes for computing the set of all-pairs shortest paths. We tried various values of *k* and found that the centrality distribution started to stabilize around 100 pivots, which we used as the value of *k* for all our experiments.
5. **Current flow betweenness centrality**: Current-flow betweenness centrality, proposed by Newman in 2005 [29], uses an *electrical current model* for information spreading in contrast to betweenness centrality (which uses shortest paths). Because of its high complexity, we use an approximation algorithm proposed in [26] that is able to approximate the true value within absolute error of a parameter *ϵ* with high probability and has run-time $O(\left(1/\epsilon^{2}\right)m\sqrt{k}log\left(n\right))$, with *m* and *n* being the number of edges and nodes respectively. We use the default *ϵ* value of 0.5 in the NetworkX implementation of this algorithm. Another parameter *k*_*max*_, which is the maximum number of sample node pairs to use for approximation was set to 10,000.

While other centrality measures also exist, we chose the five measures enumerated above for various reasons. First, all five measures are evaluated and have been used in many publications over the years, which allow a stronger basis for comparisons. An advantage of being more established is that standard implementations for these also exist in packages like Python NetworkX, which would allow our presented results to be more easily replicated for other interested researchers. Second, the five measures are also *diverse*. While betweenness and degree centralities are classic measures that (respectively) capture the importance of a node from an information-flow standpoint and local connectivity respectively, more modern centrality measures such as current flow betweenness centrality are inspired by more advanced models (such as the electrical current model), often from the natural sciences. The theoretical properties of these measures have also been much discussed in several reviews and surveys, as earlier described in *Background and Related Work*. Beyond these given measures, however, we note that re-running these experiments with other centrality measures, including the modular centrality and the overlapping modular centrality, is an interesting avenue of future work that may yield further insights into the networks.

One methodological concern that might arise when using such a range of centrality measures is that their statistics may not be directly comparable. We address this issue in two different ways. First, when reporting on these measures, we use a range of statistics, as opposed to just means and standard deviations. For example, by also reporting on minimum and maximum observed values, we provide an accurate sense of the scaling properties of these measures (at least, relative to one another). Second, when measuring *associations* between these measures, we use the non-parametric Spearman’s rank-order correlation, rather than measures like the Pearson correlation, which only tend to work for linearly related data. We provide additional details on the rationale for using Spearman’s correlation subsequently, but one important advantage is that it allows us to compare the centralities as *ordinal* variables. We emphasize that we do not claim that any one centrality measure is more or less inferior (or even informative) than another. Rather, as our results will show, the centrality measures (taken together) provide a more comprehensive and reliable picture of the findings than any one centrality measure could have been trusted to do.

## Results

### Research question 1: Class-specific centrality distributions

Recall that the first question sought to investigate which of the three entity classes (intermediaries, offshore entities and officers) in the Panama Papers had high centrality values compared to the others (thereby signifying higher importance of that class of entities) and also whether the centrality measures were consistent with respect to this determination. Using the centrality measures noted in the previous section, we tabulate the key results below.

First, in Table 1 we provide some basic statistics for each of the three entity classes and the five centrality measures. The results show that, in general, officers are less focal in this network compared to intermediaries and offshore entities. In looking at the mean centralities across measures and classes in Table 1, we find that, with the exception of closeness centrality where the average officer’s centrality (0.0422) far exceeds that of intermediaries (0.00787) and is only slightly lower than that of offshore entities (0.0463), the average centrality for an officer node is usually an order of magnitude lower than for intermediaries. However, the quantitative difference must be interpreted carefully, as prior work has shown that centrality measures are best used in a ranking-based framework (i.e., for ranking the nodes in order of importance) rather than for quantifying the importance (or the differences in importance) [30]. A less drastic, but still highly significant, difference is observed between the centralities of offshore entities and intermediaries. Even when considering the most central (*Max*.) entity in each class, we still find the highest value to be obtained by an intermediary in most cases, though the betweenness centrality is an interesting exception, in that the most central officer (0.320) achieves a much higher value compared to the most central intermediary (0.0627) or offshore entity (0.0146). Even these basic statistics, therefore, show that interpretations of entity importance can start diverging depending on both the entity class and the specific centrality measure used.

**Table 1:** Centrality maximum, mean and standard deviations observed for (from top to bottom) intermediaries, officers and offshore entities.

	D	I	C	B	F
Max.	**0.0151**	**1.0**	0.0965	0.0627	**1.725**
Mean	**3.259e-5**	**0.399**	0.00787	**8.741e-5**	**0.315**
Std. Dev.	**2.59e-4**	**0.409**	0.0213	**1.25e-3**	**0.435**
Max.	8.33e-3	0.4	**0.115**	**0.320**	0.875
Mean	2.64e-6	7.6e-3	0.0422	3.87e-6	8.67e-4
Std. Dev.	1.812e-5	0.0318	0.0298	6.66e-4	0.0138
Max.	2.16e-3	**1.0**	0.103	0.0146	1.575
Mean	5.089e-6	0.0329	**0.0463**	1.026e-5	0.0294
Std. Dev.	8.335e-6	0.147	**0.0329**	1.68e-4	0.141

D, I, C, B and F stand respectively for degree, information, closeness, betweenness and current flow betweenness centrality respectively (as defined in in the text). For all rows, the highest value among the three entity classes (i.e. across sub-tables for that column) is in bold.

With this methodological caveat in mind, a key result that does emerge in Table 1 is that intermediaries are significantly more central than the other entity classes. This result is not particularly surprising, as there is some evidence that centrality and intermediary-like function is correlated (at least in some systems, such as transportation) [31]. Also, as far back as 1978, Freeman [4] argued that central nodes were in the ‘thick of things’ or were the sort of focal points or gatekeepers that intermediaries play in complex systems involving finance, auditing and law.

To study the relationship between the centrality measures after controlling for the entity class, we quantify (in Table 2) correlational relationships, using the non-parametric Spearman’s rank-order correlation, between the different centrality measures. Note that, except for the small negative correlation between information and degree centrality in the offshore entity correlation table (*p* = 0.0106; hence, significant, but not highly significant), we found all results to be highly significant (*p* ≤ 0.01). The main reason that Spearman’s rank correlation is preferred as an associative metric for these experiments is that the different centrality measures have non-intuitive and non-homogeneous *scaling* and the relationship between them may not be linear, which would lead to a methodological issue with using measures like the Pearson’s correlation. However, since all the variables are *ordinal* (even if their statistical and scaling properties are different), we can measure the monotonic relationship between them to understand how they co-vary across both entities and entity classes. Hence, the Spearman’s rank correlation is the methodologically appropriate measure to use here.

**Table 2:** Centrality correlations (using spearman rank correlation) of the intermediary, officer and offshore-entity nodes in the Panama network (in that order, top to bottom).

	D	I	C	B	F
D	1.0	-0.694	0.812	0.466	0.833
I	-0.694	1.0	-0.969	-0.500	-0.432
C	0.812	-0.969	1.0	0.515	0.587
B	0.466	-0.500	0.515	1.0	0.030
F	0.833	-0.432	0.587	0.030	1.0
AVG.	0.483	-0.319	0.389	0.302	0.404
D	1.0	0.125	0.075	0.523	0.868
I	0.125	1.0	-0.483	0.058	0.201
C	0.075	-0.483	1.0	0.100	-0.023
B	0.523	0.058	0.100	1.0	0.456
F	0.868	0.201	-0.023	0.456	1.0
AVG.	0.518	0.180	0.134	0.427	0.500
D	1.0	0.006	0.171	0.718	0.562
I	0.006	1.0	-0.479	-0.305	0.376
C	0.171	-0.479	1.0	0.516	-0.087
B	0.718	-0.305	0.516	1.0	0.205
F	0.562	0.376	-0.087	0.205	1.0
AVG.	0.491	0.120	0.224	0.427	0.411

Column-wise averages are also noted. Statistical significance of the results is noted in the text.

The results show that, *on average*, with the exception of the information centrality for intermediary entities, there is reasonable positive correlation between all the centrality metrics, though the values vary considerably, and for certain cases, there are negative (sometimes, strongly so) correlations. For example, there is a small (but significant) negative correlation between the closeness and current-flow betweenness centrality distributions of both officers and offshore-entities, but the same correlation becomes positive when considered for intermediaries. This lends further credence to the hypothesis that we cannot study structure in the Panama Papers without controlling for either a centrality measure used or the class of entities being studied.

Furthermore, since the diagonal correlations are guaranteed to be 1.0, we can subtract 0.25 from each average if we do not wish to consider the diagonal, but only the correlations between distinct centrality measures. When only averaging off-diagonal elements for each column, we find that *I* becomes negative for all three entity classes, while *C* becomes negative for officers and offshore entities (and only barely positive for intermediaries). Yet, *I* and *C* are negatively correlated in all three entity classes.

In the *Background and Related Work* section, we mentioned that Amrit and Maat [16] had determined (with simulated information flows) that information centrality was more similar to degree (and also eigenvector, which is not considered in this work) than to closeness centrality. In Table 2, we find this to be partially true (*I* is always negatively correlated to *C*) although *I* is also negatively correlated with *D* for intermediaries, and very weakly correlated with *D* for offshore entities. This suggests that, even structurally, different kinds of information are flowing (with different strengths) between entities in these different classes, and a good theory would need to robustly explain such varying associations.

Even though intermediaries are the most central entities in the network, they also seem to have the highest variance (typically) in Table 1. This suggests significant *distributional* differences *between* entity classes, even after being conditioned on a single centrality measure.

**Fig 2:**
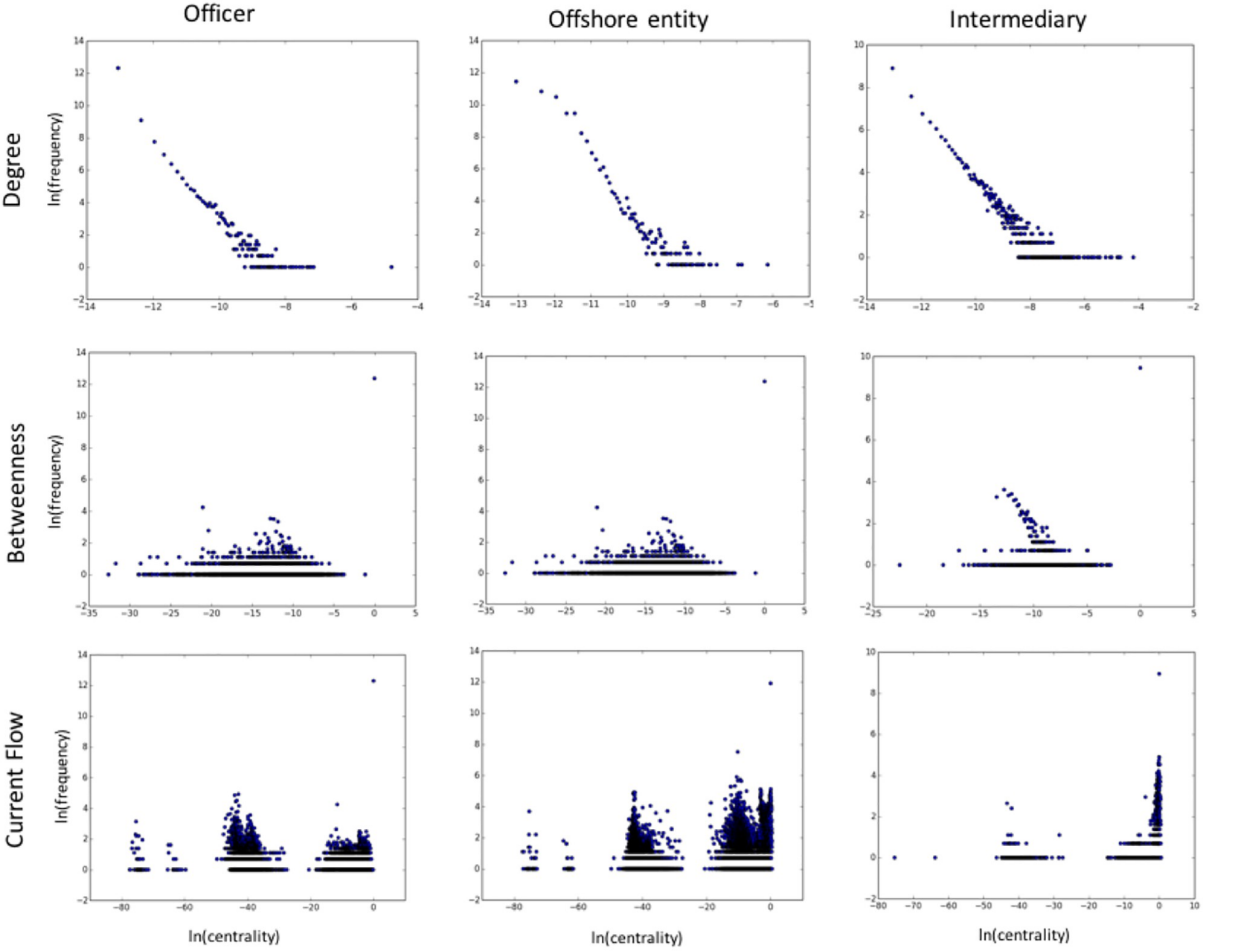
Degree, betweenness and current flow centrality frequency distributions. Both axes are on the natural log scale. Note outliers at *x* = 0 for both current flow and betweenness.

**Fig 3:**
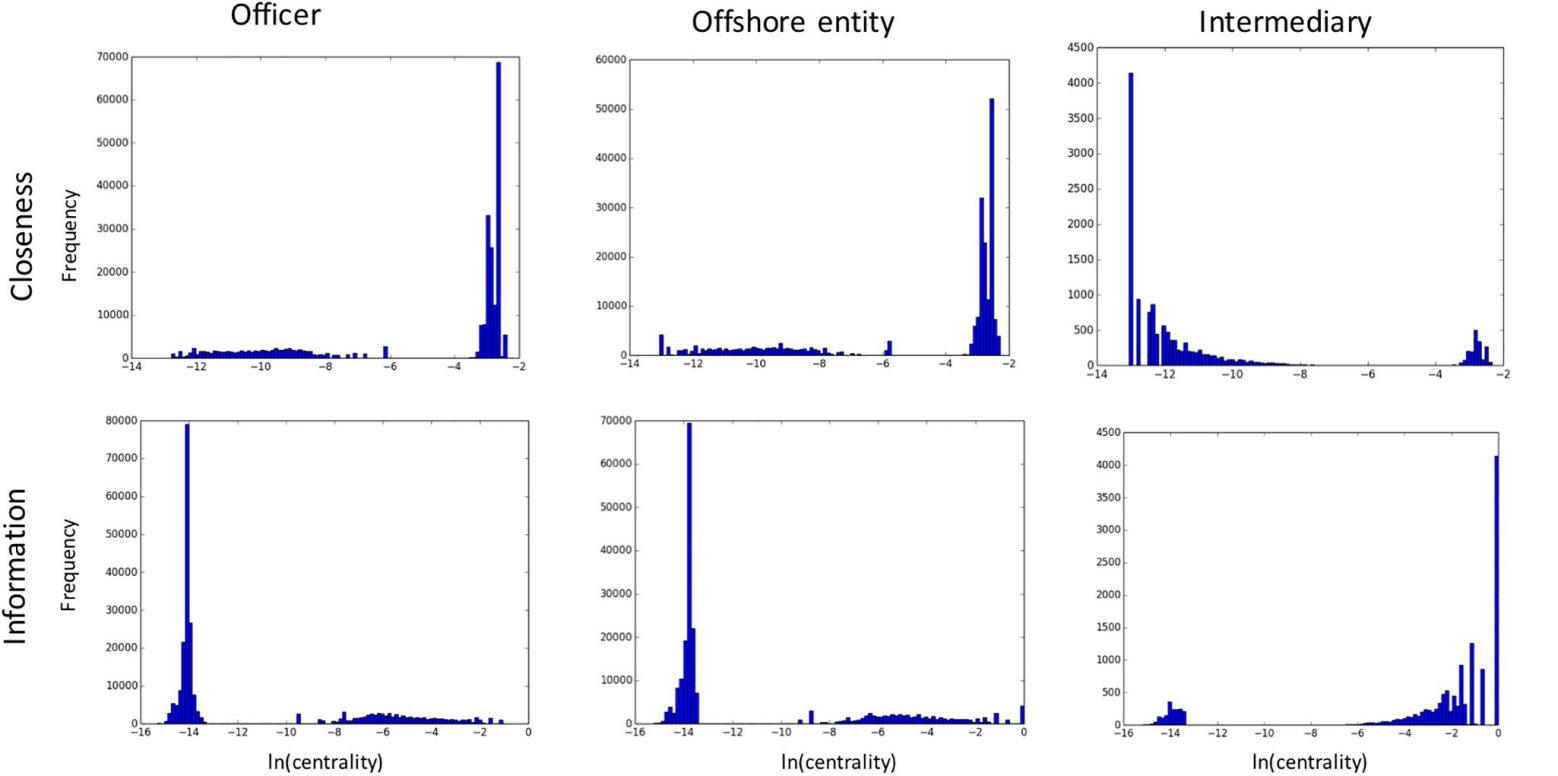
Closeness and information centrality frequency histograms. Only the x-axis is on the natural log scale, with a granularity of 500 bins per plot.

However, although useful, correlational, point-statistical and aggregate statistical measures only provide limited information about the actual centrality distributions of the entity classes. To understand the distributions of class-specific centralities, we computed histograms of centrality values for all three entity classes and all five centrality measures. Fig 2 illustrates these histograms for *degree*, *betweenness* and *current flow* centralities, while Fig 3 illustrates the histograms for *information* and *closeness* centralities. We separated these plots based on the observed extremity of values on the y-scale. For example, while the x-axis is plotted on a natural log scale (with ln(0) taken to be 0) for both figures, Fig 2 also uses the log scale for the y-axis (since there are extreme differences that are difficult to illustrate using an ordinary scale). In comparison, Fig 3 has less extremity and the trend is better illustrated using a histogram, with the y-axis (for a bin) now defined as the count of nodes having centralities in the bin range. Formally, looking at the data in Table 1. we note that information and closeness centralities generally have smaller standard deviation as a percentage of the mean, compared with degree and betweenness centralities. Current flow seems to be more like the former, but has an extreme outlier at 0, as shown in Fig 2.

There is a clear difference in the conclusions that one would draw from these two figures, notwithstanding class differences within the context of a single centrality measure. Particularly prominent is the clear difference exhibited by the centrality frequency distribution for intermediaries compared to the other two classes, for both the information and closeness centralities. The actual relationships seem to be inverted when comparing *across* the two centralities, lending further credence to previous observations that the information centrality is expressing a ‘different’ model of importance than the other measures. In contrast to all other centralities, the degree centrality exhibits a stable (and relatively homogeneous) power-law distribution for all three entity classes. Current flow and betweenness centralities have more heterogeneity, but do not show the drastic differences between classes as do the closeness and information centralities.

These differences also raise the question as to whether the trends being shown in the figures for a *particular* centrality measure would *reverse* for a particular entity class compared to the *overall* network. That is, if we computed the centrality frequency plot for the full network, rather than ‘separating’ centrality results by entity class as we have done in these results, would the actual conclusion (about the positive or negative correlation observed in such a trend) be inverted? Such an inversion would be an important instance of *Simpson’s paradox*, also called the *Yule-Simpson effect* (see [32] for a review on this effect in research findings), and would provide strong evidence for always being wary, at least in the context of the Panama Papers but possibly beyond, of the validity of such findings without controlling for the centrality measure being used *and* the entity class being studied.

To quantify the extent of Simpson’s paradox for a particular centrality measure, we compute the Spearman’s rank correlation between two paired variables, namely the centrality and the frequency of that centrality. We compute the correlation both for the individual entity classes (per centrality measure) as well as for the overall network. Table 3 shows the *sign agreement* between the former and the latter. A negative sign indicates Simpson’s paradox for that particular entity class. For intermediaries, we find that the paradox manifests for current flow centrality, which is not apparent from Fig 2, where the three plots seem to be complementing each other. Furthermore, we observe the paradox for offshore entities on the betweenness centrality, which is also not apparent in Fig 2. These results show that the centralities seem to be capturing very different phenomena about these entity classes and their interactions than suggested by the overall network or the centrality behavior of the other entity classes. We do not have a sociologically grounded or theoretical explanation for what may be causing such reversals in the Panama network, but believe that it is an important characteristic of the network, especially given its unusual nature.

**Table 3:** Sign agreement (with + indicating agreement and - indicating disagreement) between the spearman rank correlation coefficients (computed between centralities and their frequencies) of the overall network and a specific entity class.

	D	I	C	B	F
Intermediary	+	+	+	+	-
Offshore Entity	+	+	-	+	+
Officer	+	+	+	+	+

### Research question 2: Jurisdictional dependencies

Thus far, we have studied the entities in the Panama Papers from a global perspective. In the real world, these entities are heavily constrained (or encouraged) by their national jurisdictions; in many cases, they are set up to specifically take advantage of their tax jurisdictions for their clients. The popular notion (often also depicted and dramatized in fictional works) is that intermediaries and offshore entities are set up in ‘tax havens’ such as the Cayman Islands. In practice, complex multi-hop chains of entities are involved in moving money from the originator to its presumptive final destination. Rather than take on the daunting task of uncovering or discovering such potentially illegitimate chains of transactions (which may not even be possible from the public record), we take on the more modest goal of measuring national-level jurisdictional dependencies of the three entity classes. In other words, we are looking to see if national jurisdictions demonstrate non-random patterns in the class-controlled centrality distributions of the entities within the jurisdiction, as well as inter-centrality (and inter-class) correlations between the entities.

**Fig 4:**
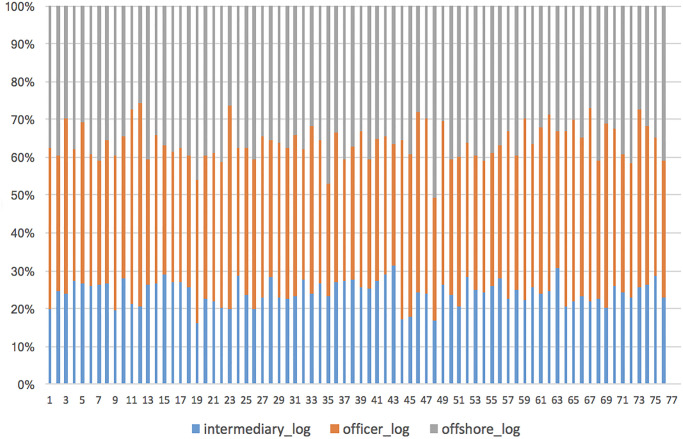
Share of entity class versus country index. A log-transform was done on the count of entities per country per class, and country indices were assigned arbitrarily. Hence, each point on the x-axis represents a single country, with the entity class shares shown as y-value percentages.

As a first step towards such an analysis, we obtain the jurisdiction of each node in the network. Since the jurisdictions of some nodes are unknown, and in some cases, the jurisdiction is also ambiguous or complex (more than one jurisdiction is listed), we only retain nodes that are associated with exactly one jurisdiction. Furthermore, to avoid the effects of jurisdictions that do not have sufficient representation (i.e. too few entities have that jurisdiction), we limit our analysis to jurisdictions that have at least ten associated entities from each of the entity classes. Following this preprocessing, we are left with 320,564 entities and 76 jurisdictions for our study. The probability distribution of country frequency for all three entity classes is plotted in Fig 4. The average number of intermediary, officer and offshore entities per country is 153.6, 1626.4 and 2437.9 respectively.

Returning to the core of the research question, we gathered a detailed set of correlations to determine the association between centrality and nationality for all entity classes. Before describing our experimental design, we note that, since the associations are measured at the level of countries, we have to assign an ‘importance score’ to a country in the context of a given centrality measure and a given entity class. The simplest way to assign such a score (and one that we adopt in this paper) is to compute the mean of the centralities (for the given centrality measure) of all entities that belong to the given entity class and that list the country as their jurisdiction. Recall that our earlier preprocessing filtered out countries that do not have at least ten entities (listing that country as their jurisdiction) from each of the entity classes to ensure sufficiently robust statistics for all entity classes. Put more formally, given the centrality *C*(*u*) of node *u* that is associated with national jurisdiction *J* and has entity class *E*, we define *u*(*j*) = *J* and *u*(*e*) = *E* in a slight abuse of notation. The importance score $I_{C}^{E}\left(J\right)$ of *J* is then given by:

$$\begin{array}{c} I_{C}^{E}\left(J\right)=\frac{\sum_{\left\{u|u(j)=J,u(e)=E\right\}}C\left(u\right)}{\left|\{u|u(j)=J,u(e)=E\}\right|} \end{array}$$

We use the subscript *C* on *I* to indicate that the importance score depends on the centrality measure employed. Similarly, we use the post-script *E* to indicate the class of entities (e.g., intermediaries) being used. In designing the experiments for such an analysis, one might be tempted to only measure correlations within the context of a *single* centrality measure (i.e. limit the scope of the study to a question such as *how strong or significant is the association between the country importance scores of intermediaries or officers (or any pair of distinct entity classes), with importance scores computed using a single (given) centrality measure such as betweenness?*). However, as we saw earlier, there are non-trivial relationships between the different centrality measures both within and across entity classes, and it is certainly plausible that such relationships may persist (or become amplified) when we conduct a similar analysis at the level of countries. Hence, we do not impose a constraint on the centrality measure used or the entity class. Rather we consider all five centrality measures for this research question as well; hence, there are five importance scores calculated per jurisdiction per entity class.

**Fig 5:**
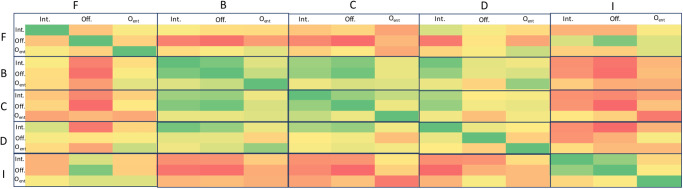
Spearman’s rank correlation coefficients between each pair of 76-dimensional importance score vectors, defined in the text. Similar to previous figures, *F, B, C, D* and *I* refer to *current flow betweenness, betweenness, closeness, degree* and *information* centralities respectively, while *Int., Off* and *O*_*ent*_ respectively stand for *Intermediary, Officer* and *Offshore Entity*. Yellow values indicate weak (or no) correlation, green values indicate high positive correlations and red values indicate high negative correlations.

For our analysis, we begin by computing a 15 × 15 matrix (i.e., 3 entity classes × 5 centrality measures) of Spearman’s rank correlation coefficients between each pair of 76-dimensional importance score vectors $I_{C_{i}}^{E_{i}}\left(J\right)$ and $I_{C_{j}}^{E_{j}}\left(J\right)$ (there are 76 dimensions because there are 76 jurisdictions in our filtered dataset described earlier), where *C*_*i*_, *C*_*j*_ (∈ {*D*, *C*, *B*, *I*, *F*}) and *E*_*i*_, *E*_*j*_ (∈ {Intermediary,Officer,Offshore Entity}) are allowed to vary independently, leading to fifteen possible vectors. To illustrate effective patterns, we plot the matrix as a heat map (Fig 5). Yellow values indicate weak (or no) correlation, green values indicate high positive correlations and red values indicate high negative correlations.

We find from the heatmap that:

1. Analogous to our findings for Research Question (RQ) 1, information centrality (*I*) is once again found to be negatively or weakly correlated to closeness centrality (*C*), and also to betweenness centrality (*B*). Unlike the results in Table 2 however, we do find mostly negative correlations to degree centrality (*D*) as well. One hypothesis is that information centrality may exhibit Simpson’s paradox-like behavior when using jurisdiction (rather than entity class, as in RQ1) as a control variable. We leave exploring this hypothesis for future work.
2. Betweenness centrality is generally the most positively correlated, both with itself (among the different entity classes) and also with many of the other centrality measures. This suggests that, for general studies of structure and influence on the Panama Papers, *B* may be a more reliable metric than others.
3. Interestingly, when we look at the ‘diagonal’ blocks in the heatmap, we find that, for both *D* and *F* (current-flow betweenness) there is weak or even negative correlations between entity classes. These centralities seem to be suggesting that different jurisdictions occupy different niches, since (using *F* as an example), high intermediary centralities would suggest weaker offshore entity centralities. Similarly, for degree centrality, jurisdictions’ offshore entity centralities are negatively correlated with officer centralities.

## Discussion

We summarize some of the key implications of the results in the previous sections:

1. First, while differences are observed when using different centrality measures, an important commonality (with few exceptions) is that entities that are intermediaries are seen to have overwhelmingly high centrality compared to both officers and offshore organizations. Intermediaries clearly play a crucial role in the financial system of money-movement represented in the Panama Papers. Since intermediaries tend to be incorporated locally (such as a law firm or an accounting firm), they would be subject to that locality’s jurisdiction where a shell corporation is being created or money is being moved, as opposed to a multinational corporation that may be subject to the source-country’s jurisdiction.
2. Second, despite the finding above, the choice of centrality is an important one, since some centralities yield inverted results compared to the others. In particular, the information centrality is often negatively correlated with the other measures; in some cases, the current-flow betweenness centrality and the closeness centrality can also exhibit inverted behavior. Most likely, it is not the case that one centrality measure is ‘right’, but instead, we hypothesize that the different centralities are capturing different kinds of importance. Previous work has shed some light on this [4], but the issue is not resolved in the broader community.
3. Third, Simpson’s paradox-like behavior is observed when we attempt to understand and compare the relation between centrality values and their frequencies at the overall network-level (which includes all the entities and classes) versus for each of the classes individually. The behavior is observed when using the current-flow betweenness and closeness centralities for intermediaries and offshore entities respectively. For the majority of centralities, there is agreement. More research is needed to understand why the paradox arose for the two cases above.
4. Finally, many of the results above are replicated when we account for jurisdictions. In particular, information centrality is again found to exhibit somewhat inverted behavior. However, clear differences also start to emerge once we look at jurisdictions, and the results suggest that different jurisdictions or nations may serve as different niches in the system.

An important point is that centrality measures, though well established in the network science and complex systems community as a means for quantifying importance, are not perfect. There is also evidence to suggest that they may under-estimate the importance of non-hub nodes [33]. Some of these caveats likely apply to the Panama Papers, which is an unusual system to begin with, and which has many fragmented components. As the influential review of Borgatti and Everett showed, the accuracy of centrality indices also depends on the network topology [34].

## Conclusion

Since their release, the Panama Papers have come under broad scrutiny by experts in legal scholarship, sociology and tax policy. A limited number of studies have used computational techniques to study the structure of the entities and officers in the network, but studies spanning jurisdictions or entity classes have thus far not been forthcoming. In this paper, we specifically study entity importance across three different classes (intermediaries, officers and offshore entities) in the Panama Papers by employing five well-defined centrality measures. Our experimental study is based on two related research questions, one of which maps out consistencies and differences (in measuring entity importance) between the centrality measures and entity classes, and the second of which considers similar questions but at the aggregate view of national jurisdictions.

Our results suggest many open questions for future investigation. Although we studied the Simpson’s paradox in the context of measuring centrality frequency correlations, a similar experiment could be designed where the national jurisdiction is used as the control variable. We hypothesize that values for this variable for which Simpson’s paradox manifests may be correlated with legal and regulatory characteristics (such as whether the jurisdiction is a tax haven, scores badly on corruption indices etc.) of the jurisdiction. Similarly, in the context of Research Question 2, we note that the centralities of the various entity classes *within* a national jurisdiction can also be studied using a similar experimental framework. In principle, such experiments are similar compared to those conducted in support of Research Question 1, but limited to the set of entities {*u*|*u*(*j*) = *J*} given a particular jurisdiction *J* (e.g., the USA). Heatmaps could be constructed per country, and the jurisdictional properties of countries could be studied comparatively by studying individual heat map differences. Finally, we also plan to look at the community structure at both the local and aggregated levels to examine similar and dissimilar behaviors, in a similar vein to [20]. The core-periphery structure of the network using hierarchical measures (similar to [22]) is yet another relevant aspect of future work, since identifying how nodes are positioned in the Panama Papers network could provide useful insights.
